# Fluorescent nanosensors reveal dynamic pH gradients during biofilm formation

**DOI:** 10.1038/s41522-021-00221-8

**Published:** 2021-06-17

**Authors:** Birte Hollmann, Mark Perkins, Veeren M. Chauhan, Jonathan W. Aylott, Kim R. Hardie

**Affiliations:** 1 Biodiscovery Institute, School of Life Sciences, University Park, University of Nottingham, Nottingham, UK; 2 Advanced Materials & Healthcare Technologies, School of Pharmacy, University of Nottingham, Nottingham, UK

**Keywords:** Biofilms, Pathogens

## Abstract

Understanding the dynamic environmental microniches of biofilms will permit us to detect, manage and exploit these communities. The components and architecture of biofilms have been interrogated in depth; however, little is known about the environmental microniches present. This is primarily because of the absence of tools with the required measurement sensitivity and resolution to detect these changes. We describe the application of ratiometric fluorescent pH-sensitive nanosensors, as a tool, to observe physiological pH changes in biofilms in real time. Nanosensors comprised two pH-sensitive fluorophores covalently encapsulated with a reference pH-insensitive fluorophore in an inert polyacrylamide nanoparticle matrix. The nanosensors were used to analyse the real-time three-dimensional pH variation for two model biofilm formers: (i) opportunistic pathogen *Pseudomonas aeruginosa* and (ii) oral pathogen *Streptococcus mutans*. The detection of sugar metabolism in real time by nanosensors provides a potential application to identify therapeutic solutions to improve oral health.

## Introduction

The biofilm communities that bacteria form on surfaces are dynamic and complex. The architecture of biofilms and the identity of extracellular components have been characterised in vitro for a variety of biofilms. Although it is not yet clear how representative this is of in vivo bacterial communities^1–5^, further in vitro investigation of dynamic biofilms will help deliver the tools required to interrogate the emerging more realistic models. While there is evidence for fluid channels and microcolonies within in vitro biofilms, the characterisation of the environmental microniches these create has been limited. Although a number of analytical tools have been applied to static biofilms, for real-time monitoring, tools that are suitably sensitive at the required resolution now require testing in appropriate flow biofilm models^6–16^. In this article we demonstrate the application of fluorescent nanosensors capable of dynamic pH monitoring of the environmental microniches within biofilms using two important pathogens.

Firstly, we selected the Gram-negative bacterium *Pseudomonas aeruginosa*, an opportunistic pathogen, causing infections in immunocompromised patients, including those suffering from burns, wounds and cystic fibrosis (CF)^17–20^. These infections are challenging to treat, due to the intrinsic antibiotic resistance of *P. aeruginosa*, which contributes to *P. aeruginosa* infections being the leading cause of morbidity for CF sufferers. *P. aeruginosa* is prevalent in the environment creating numerous potential reservoirs of infection. *P. aeruginosa* exerts its pathogenicity through the production of several factors, including those shown to play a key role in biofilm attachment such as extracellular DNA^21–24^. Biofilm formation by *P. aeruginosa* is particularly important for it to establish chronic infections and has therefore been studied intensively.

Secondly, we chose the Gram-positive oral pathogen *Streptococcus mutans*. Oral biofilms play a crucial role in the aetiology of oral diseases, such as dental caries and periodontitis, which can lead to increased economic burden and reduced quality of life^25^. Oral biofilms are characterised by a bacterial shift from early colonisers of the tooth towards increasingly acid-producing (acidogenic) and acid-tolerant (aciduric) species^26^. *S. mutans* is both an aciduric and acidogenic bacterium and a pre-dominant species in late stage oral biofilms^27,28^. The interest in oral bacteria such as *S. mutans* lies in their participation in dental caries, the most common infection affecting humans, through the production of organic acids^29^. Initially, *S. mutans* uses sucrose as a substrate to synthesise glucan via glucosyltransferases, in order to provide anchoring sites on the tooth’s enamel. This enables bacteria to continue to colonise and establish more biofilm^30–32^. Once established, *S. mutans* ferments available carbohydrates, readily found in a sugar-laden diet, which results in the production of organic acids^33^. This acidification leads to the formation of dental caries^34–36^. Robust, well-characterised biofilm models exist for both *P. aeruginosa* and *S. mutans*, making them an ideal choice for this study.

An environmental characteristic unlikely to be static and homogeneous in biofilms is pH, making it an attractive option to quantify spatially in real-time. It is also important to characterise the environmental pH of microorganisms because it can influence many different physiochemical properties such as polymer–polymer, ion–polymer and macromolecule–polymer interactions^37^. Furthermore, the formation of chemical gradients (such as in pH, redox potential and ions) within the biofilm community can provide indications about microbial metabolism^38–42^.

The relationship between pH and biofilm formation differs, depending upon species. As outlined above, acidification within oral biofilms containing *S. mutans* occurs during development of dental caries, and an ability to adapt to this change in pH is key to the virulence of this pathogen. To thrive, *S. mutans* mounts the acid-tolerance response (ATR) to maintain an intracellular pH more alkaline than the environment in addition to modifying its cell membrane composition^43^. Like many other bacteria, some of the molecules which *P. aeruginosa* produces, such as signal molecules for quorum sensing (QS) to mediate population density-dependent cell–cell communication, are *N*-acylhomoserine lactones (AHLs). AHLs have been implicated in biofilm formation for many bacterial species. For instance, Yates et al.^44^ showed that the lactonolysis of AHLs is pH dependent, with ring closure only taking place at reduced pH levels, whereas Hostacka et al.^45^ demonstrated an accelerated biofilm production for *P. aeruginosa* at pH 7.5 and pH 8.5 in comparison to pH 5.5 using crystal violet quantification. A similar behaviour was described by Stoodley et al.^46^ who observed that *P. aeruginosa* biofilm thickness fell to 68% at a low pH of 3.

In contrast, studying the Gram-positive bacteria *Staphylococus aureus* and *Staphylococus epidermidis*, Nostro et al.^47^ found biofilms were 2.5–3 times thinner at high pH levels, suggesting this to be a possible way to control biofilm formation in this species. Studies of *Streptococcus agalactiae*, whose infection can result in meningitis, have shown an accelerated biofilm formation in highly acidic conditions^48^. For other group B streptococcal isolates, the same behaviour was found in vaginal infections of pregnant women where clinical isolates showed a higher biofilm formation rate at a vaginal pH of 4.5 in comparison to pH 7 (ref. ^49^).

There are a variety of methods to measure pH, including nuclear magnetic resonance spectroscopy (NMR), microelectrodes and surface-enhanced Raman scattering^50–52^. However, those technologies are not accessible to all laboratories and the resolution of some of the methods is not yet comparable to fluorescent-based measurements^37,52,53^. Two-photon excitation microscopy can be used to measure fluorescence ratiometry or fluorescence lifetime imaging which can both be used to quantify fluorescence in a pH-dependent manner^40^. Alternatively, ratiometric dyes with a dual emission spectrum or a combination of pH-sensitive and reference dyes can be employed. These dyes can be applied in solution, or coupled to dextrans, planar surface (optodes), or particles (nanosensors)^54^. Nanosensors containing commercially available fluorophores have aroused increasing interest and are being widely implemented in approaches to pH measurement.

Nanosensors are nanoparticles that can be used for the detection and measurement of small environmental changes. Fluorescent nanosensors consist of spherical polymer particles carrying fluorophores (signal transducer) generating fast, bright responses that can be measured using real-time fluorescent microscopy^37,53,55^. Incorporation of different pH-sensitive fluorophores in the correct ratio in a single nanosensor can enable the detection of the full physiological pH spectrum from pH 3.5 to pH 7.5 (refs. ^56–58^).

A range of different pH nanosensors has already been applied in biological research, for example in cancer research where it has been suggested that low oxygen concentration and low pH increase the potential of cancer metastasis and reduce the treatment prognosis for patients with advanced stage cancers^59^. Within microbiology, silica based nanosensors were used to analyse pH microenvironments in microbial biofilms of *Escherichia coli*^53^. Hidalgo et al. could show local variation in pH from the neutral outside surface of the film to the acidic core. Nanosensors with a single fluorescent ratiometric pH-sensitive probe were used to reveal pH heterogeneity throughout a *P. aeruginosa* biofilm^37^. A slightly acidic biofilm environment was reported with a pH range between 5.6 within the biofilm and 7.0 in the bulk fluid^37^. More recently, Fulaz et al.^60^ reported the use of pH-sensitive nanosensors to detect pH gradients in biofilms, but did not extend their study to real-time analysis of the environmental microniches under flow conditions.

As different laboratories use a variety of nanoparticles, characterisation of the interaction of nanoparticles with bacteria and biofilm components is required. For their use in biological systems, it is important that the nanoparticles do not alter the metabolism and behaviour of the cells and to establish the penetration of nanoparticles into the biofilm. Penetration usually occurs by diffusion where the nanoparticles are interacting with either the bacteria or the biofilm matrix components^61–63^. The diffusion characteristics of the nanoparticles mostly depend on their size and charge but are also influenced by pore size of the biofilm, the charge and chemical gradient of the biofilm matrix, and the hydrophobicity of the environment^62–65^.

From the repertoire of nanosensors available to us, we selected polyacrylamide-based nanoparticles due to their small size. This differed from the nanosensors used by Fulaz et al.^60^, which were silica based. To enable a physiological range between pH 3 and pH 8 to be measured and visualised, two pH-sensitive fluorophores, Oregon green (OG) and 5(6)-carboxyfluorescein (FAM), were used. These fluorophores together with a reference pH-insensitive fluorophore, 5(6)-carboxytetramethylrhodamine (TAMRA), were covalently linked to a polyacrylamide nanoparticle matrix to synthesise ratiometric pH-sensitive nanosensors. To evaluate the influence of charge, both neutral and positively charged versions of the nanosensors were produced.

The penetration and interactions of the nanosensors within the biofilm were characterised, and the use of nutrient flow and time-lapse imaging enabled real-time reporting of pH modulation within a biofilm.

## Results

### Polyacrylamide nanosensors distribute extracellularly within growing biofilms

From the broad selection of nanoparticles available to us^66,67^, polyacrylamide nanoparticles were selected. The basis for this choice was to have nanosensors available that were small enough to penetrate deep into biofilms and report with the sensitivity required for high-resolution imaging as well as the option to alter surface characteristics and thereby manipulate interactions with the extracellular matrix. Polyacrylamide nanoparticles were prepared at a smaller size (34.77 ± 4.63 nm, see Table 1) to those based on silica used previously (47 ± 5 nm)^60^, and with different surface charges.

**Table 1:** Characteristics of the nanosensors synthesised in this study.

	Positively charged polyacrylamide nanosensors (*n* = 3)	Positively charged polyacrylamide nanoparticles without dyes (*n* = 1)	Neutral polyacrylamide nanosensors (*n* = 1)
Size (nm)	34.77 ± 4.63	46.41	37.51
PDI	0.168 ± 0.036	0.167	0.096
*Z*-potential (mV)	15.6 ± 3.85	17.7	−4.75

*PDI* polydispersity index, *n* number of preparations analysed.

The size, polydispersity index (PDI) and zeta potential values of positively charged polyacrylamide nanosensors, positively charged polyacrylamide nanosensors without dyes and neutral polyacrylamide nanosensors, determined by a MalvernTM DLS instrument.

**Fig. 1:**
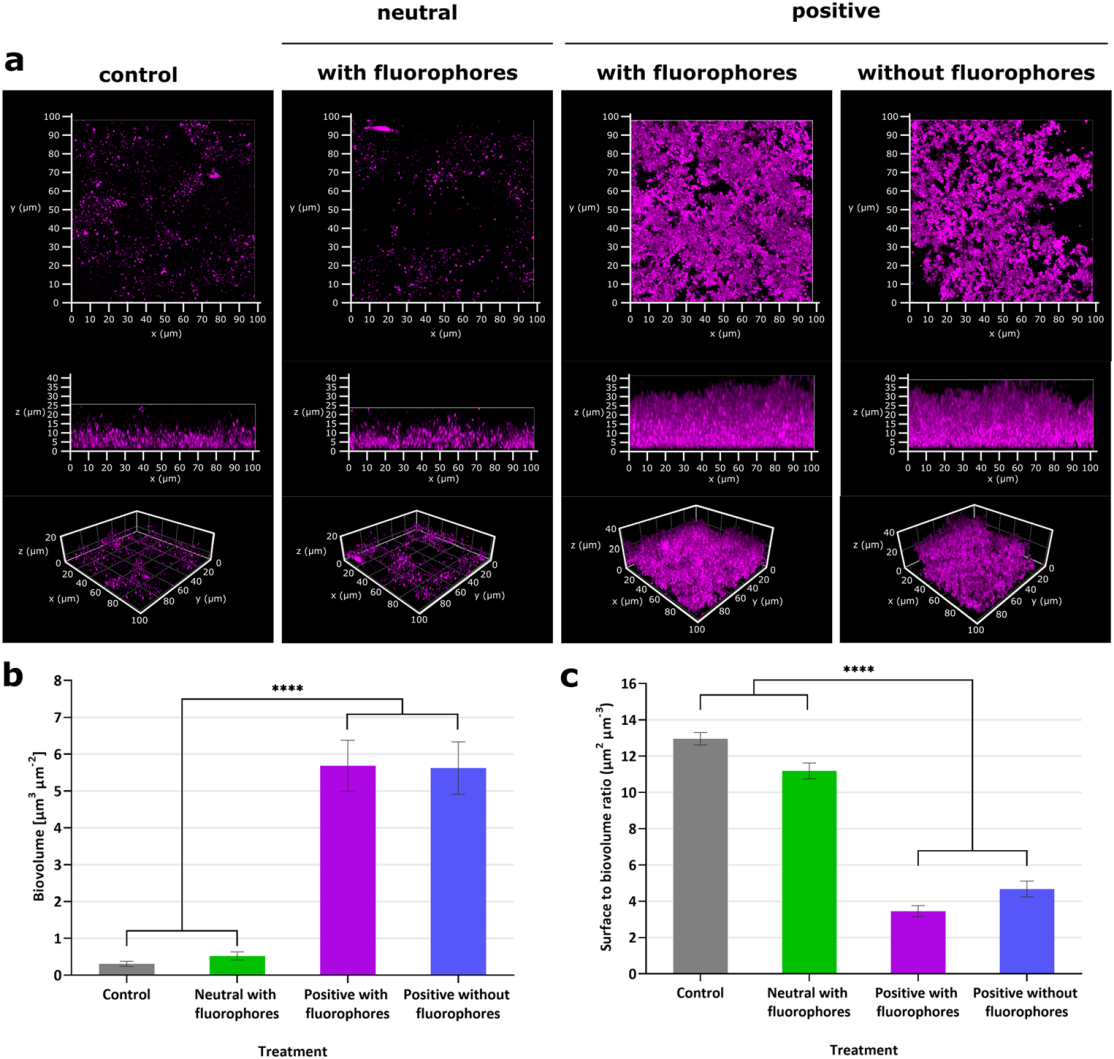
The charge of nanosensors can influence the biofilm formation of *P. aeruginosa* PAO1-N. Differently charged nanoparticles at 1 mg mL^−1^ were incubated with *P. aeruginosa* PAO1-N under static conditions. Quantitative Comstat analysis of the biofilm images were performed using ImageJ. **a** Representative confocal images of *P. aeruginosa* PAO1-N stained with CellMask^TM^ without the addition of nanoparticles (left panel), grown with neutral polyacrylamide nanosensors (left central panel), with positively charged polyacrylamide nanosensors containing fluorophores (right central panel) or with positively charged polyacrylamide nanoparticles without fluorophores (right panel). The top row is a 3D view from the top of the biofilm; the bottom row shows a 3D view from the front of the biofilm. Graphs represent **b** biovolume and **c** surface to biovolume ratio. Error bars represent standard deviation measured for different biofilm images, where *n* = 10 (control), *n* = 7 (neutral nanosensors and positive nanoparticles without dyes) and *n* = 8 (positive nanosensors), with *p* < 0.0001 represented by **** (ordinary one-way ANOVA). The same number of replicas were visually inspected.

Having settled on *P. aeruginosa* as our model system for initial optimisation experiments, strain PAO1-Nottingham (PAO1-N) was initially chosen to analyse the influence of nanosensors on planktonic and biofilm growth. The effects of nanosensors upon *P. aeruginosa* were first assessed by introducing different concentrations of nanosensors to planktonic cell cultures. To detect the effects, the growth of the bacteria was monitored via the OD_600_ every 15 min for 24 h in 96 well TECAN plates. No growth inhibition was observed for nanosensor concentrations below 25 mg mL^−1^ (Supplementary Fig. 1). In addition, super-resolution microscopy of planktonic PAO1-N grown overnight with positively charged or neutral nanosensors was performed to confirm that the nanosensors stay extracellular and to ensure that all subsequent pH measurements report on pH changes within the biofilm and the matrix rather than inside the cells (see Supplementary Fig. 2). To investigate possible effects of the nanosensors on biofilm formation, the nanosensors were suspended in the cell culture prior to the incubation with bacteria. The resultant biofilms, grown with and without nanosensors under static conditions, were analysed after 48 h using fluorescence confocal laser scanning microscopy (CLSM, Fig. 1a). Interestingly, biofilms grown together with the positively charged pH-sensitive polyacrylamide nanosensors were approximately four times thicker (40 µm) compared to biofilms grown in the absence of nanosensors or with neutral pH-sensitive nanosensors (~10 µm) (Fig. 1a). The general structure of the biofilms grown with positively charged pH-sensitive nanosensors was also notably different, showing a very dense structure. The addition of positive nanoparticles without the inclusion of fluorophores led to the similar increases in biofilm thickness and robustness as the positively charged nanosensors, suggesting that the charge of the particles was responsible for the change in the biofilm formation. Moreover, the thickness effect was dependent on the concentration of the nanosensors, with higher concentrations of 10 or 20 mg mL^−1^ reducing the thickness compared to 1 mg mL^−1^ of nanosensors (Supplementary Fig. 3).

### Polyacrylamide nanosensors rapidly penetrate an established biofilm

To investigate if nanosensors can penetrate an already established *P. aeruginosa* biofilm, nanosensors were added to a 48-h matured biofilm. Penetration of the sensors was assessed using fluorescence CLSM. To ensure that a thick biofilm could be used for the analysis, the bacteria were pre-grown with positively charged nanoparticles without fluorophores. After imaging the biofilm, positively charged or neutral nanosensors were added to the cultures and the biofilms were immediately imaged again without washing. Penetration of the biofilm by the nanosensors was assessed by tracking the fluorescence of the nanosensors throughout the thickness of the biofilm. Fluorescence within the whole biofilm was observed after 3 min following the addition of the nanosensors, suggesting that the nanosensors enter the biofilm very quickly and without obvious resistance. This behaviour was observed for both the neutral and the positively charged nanosensors.

**Fig. 2:**
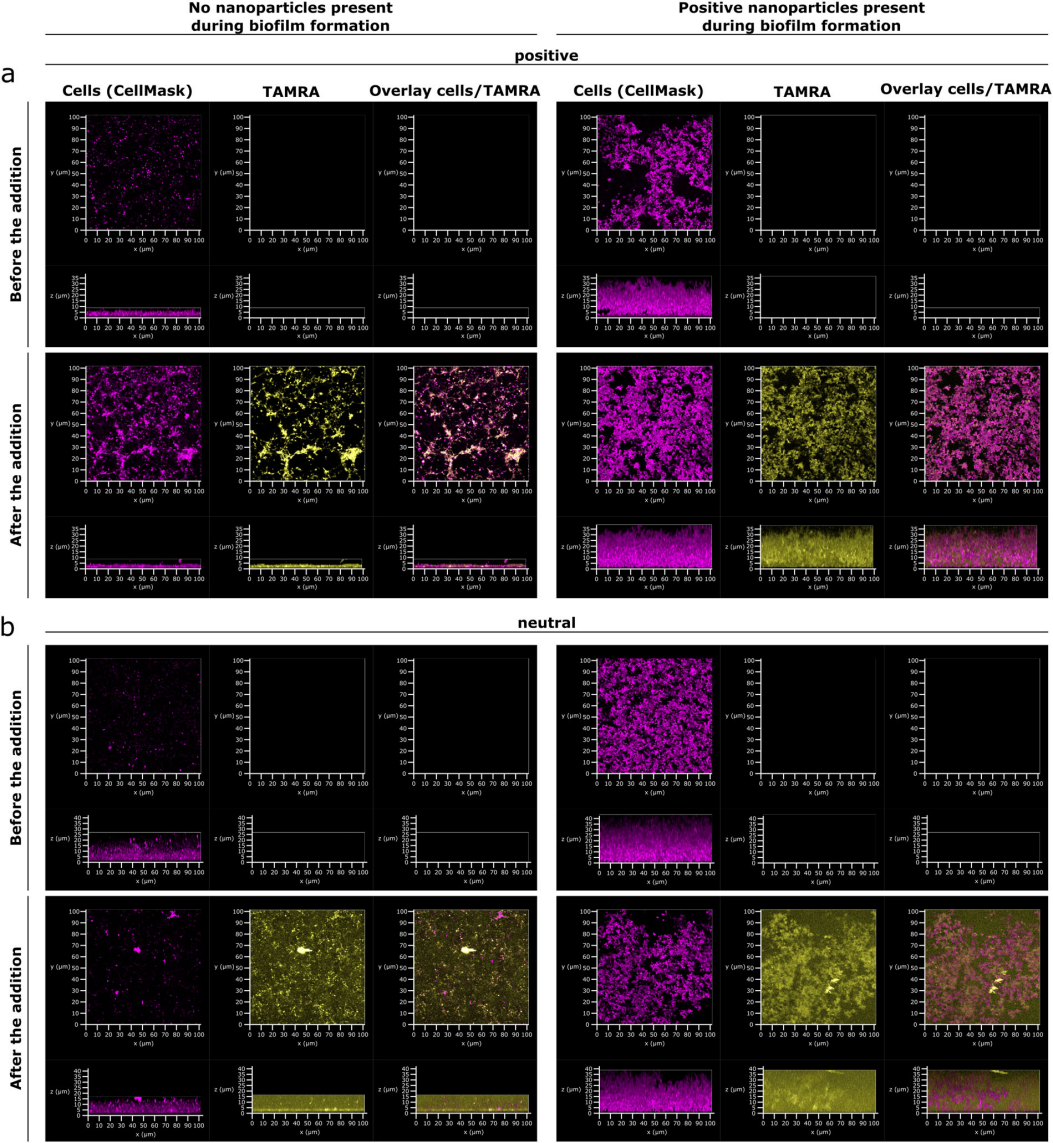
Neutral as well as positive nanosensors can penetrate established *P. aeruginosa* biofilms under static conditions. **a** Confocal images of *P. aeruginosa* PAO1-N grown without (left panel) or with (right panel) 1 mg mL^−1^ positive nanoparticles without fluorophores for 48 h. Images show cells stained with CellMask (magenta) and TAMRA fluorescence of nanosensors (yellow). For the purpose of these images TAMRA has been false coloured to yellow to facilitate visualisation of nanoparticle distribution. Top panel = before the addition of positively charged pH-sensitive polyacrylamide nanosensors. Bottom panel = after the addition of positively charged pH-sensitive polyacrylamide nanosensors to a final concentration of 1 mg mL^−1^. Left panel = **b** Confocal images of *P. aeruginosa* PAO1-N with same conditions as in **a**, but with the addition of 1 mg mL^−1^ neutral pH-sensitive polyacrylamide nanosensors.

Interestingly, when the biofilms were grown initially without the positive nanoparticles, the structure of the biofilm was disrupted when positively charged nanosensors were added but not when neutral nanosensors were added, supporting the observation that growing PAO1-N together with the positive nanoparticles results in a denser biofilm (Fig. 2).

**Fig. 3:**
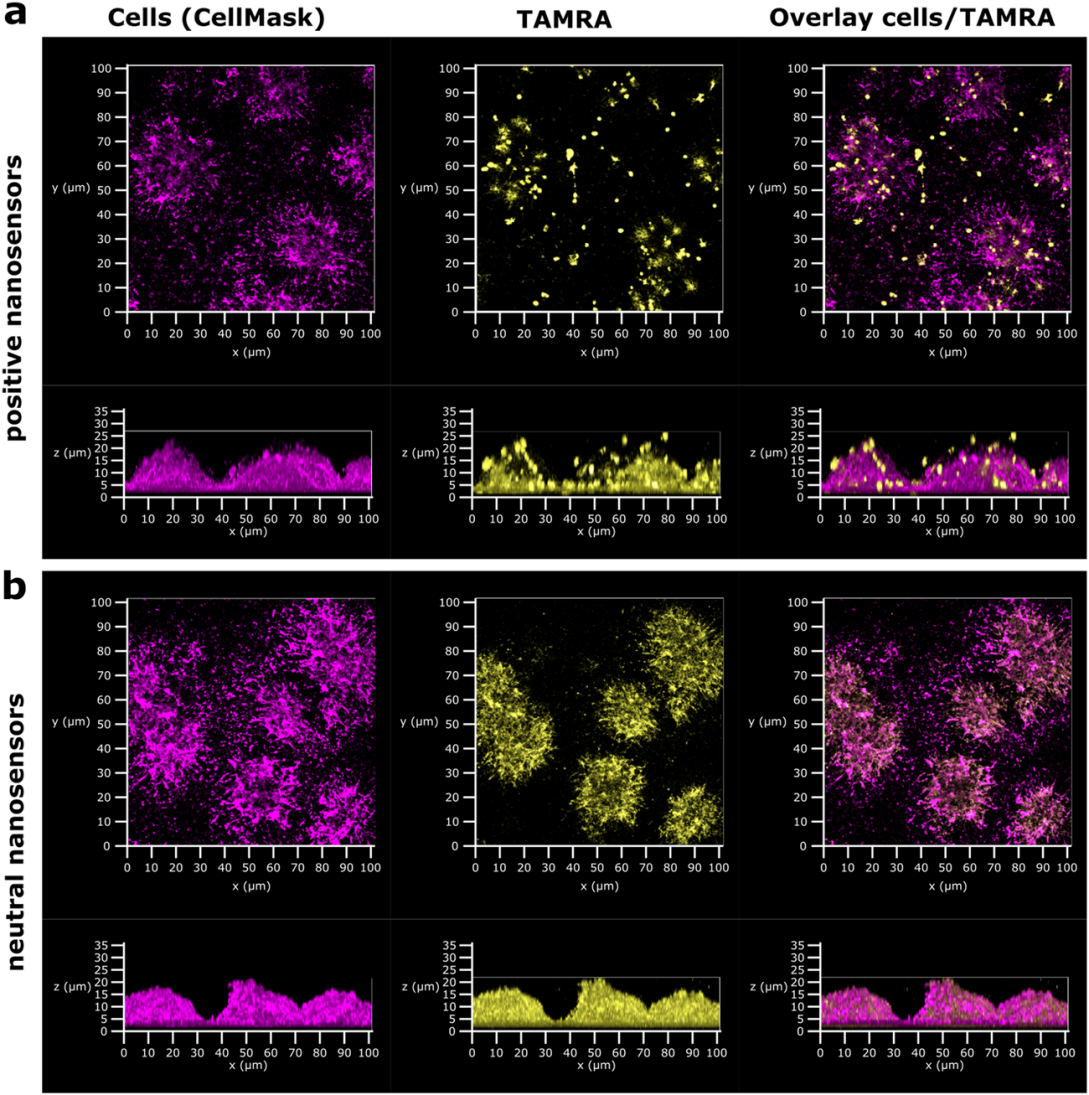
Penetration of established *P. aeruginosa* biofilms by nanosensors under flow conditions was independent of the charge of the nanosensors. *P. aeruginosa* biofilms were initially grown for 12 h without nanosensors before the medium was switched to medium with 5 mg mL^−1^ positive (**a**) or neutral (**b**) nanosensors for 5 h followed by a switch back to medium without nanosensors for another 5 h. Confocal images show nanosensor fluorescence (TAMRA, yellow) within the biofilm after the final wash step for both nanosensors (positive and neutral). For the purpose of these images TAMRA has been false coloured to yellow to facilitate visualisation of nanoparticle distribution.

In addition to a static biofilm, the flow cell BioFlux system was used to investigate penetration of biofilms by both neutral and positive nanosensors (Fig. 3). After initially growing the biofilm for 12 h in the absence of nanosensors, the inlet of the flow medium was switched to a medium containing nanosensors for 5 h followed by a switch back to medium without nanosensors for another 5 h. These timings were chosen to monitor nanosensor penetration and wash out specifically in the BioFlux. CLSM at the end point showed that there were nanosensors incorporated within the biofilm indicating penetration of the biofilm by the nanosensors. Neither neutral nor positive nanosensors changed the biofilm structure or thickness when grown within the BioFlux system.

### pH changes can be observed during biofilm formation in a flow cell system at a microcolony level using polyacrylamide nanosensors and time-lapse imaging

Time-lapse imaging together with the nanosensor technology was used to investigate pH changes during biofilm formation of *P. aeruginosa* PAO1-N over time in a BioFlux flow cell system. Images were taken every 15 min for 16 h using a Nikon widefield microscope. A red region of interest indicates a low pH, due to diminishing signal from OG & FAM under acidic conditions, whereas green to orange indicate relatively higher pH values, due to an increase in fluorescence from OG and FAM. During the first couple of hours after microcolony formation starts, the pH increases as observed by a colour shift from a more orange colour (lower pH) towards a more greenish colour (higher pH).

**Fig. 4:**
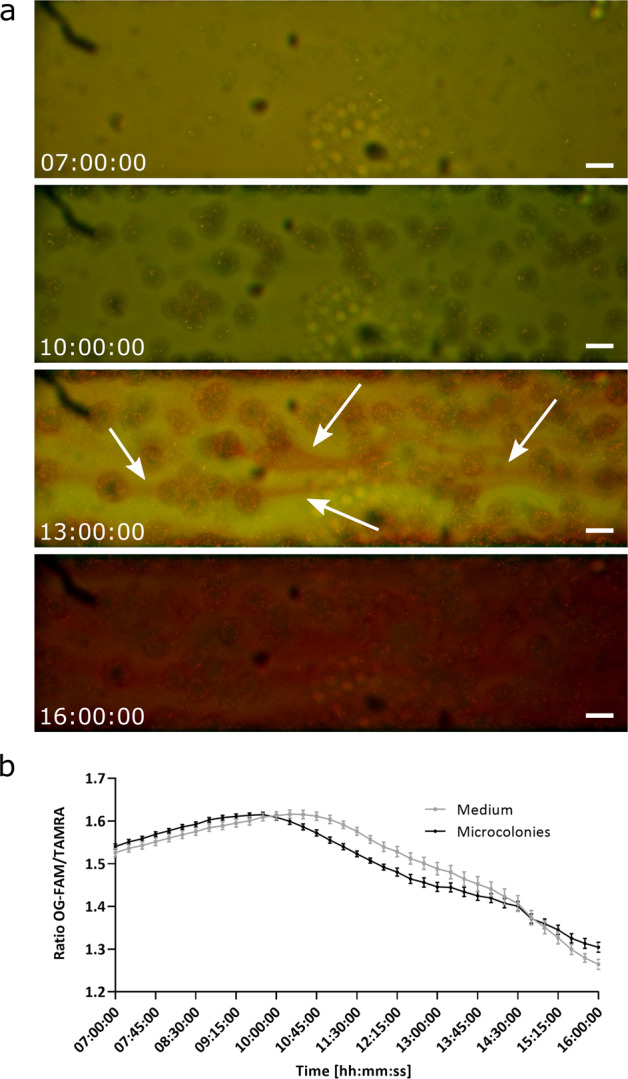
A streamer of acidic pH is evident in the downstream flow of *P. aeruginosa* biofilm microcolonies. Time-lapse images of a *P. aeruginosa* biofilm grown in a BioFlux flow cell and imaged using a fluorescence Nikon widefield microscope. **a** Time-lapse imaging of a *P. aeruginosa* biofilm grown in a BioFlux flow cell after 7 h (start of microcolony formation), 10, 13 and 16 h. Red streaks being released downstream by the microcolonies into the medium after 13 h of growth are indicated by the white arrows. Positively charged pH-sensitive polyacrylamide nanosensor were included at 5 mg mL^−^^1^ to visualise the red streaks. Scale bars represent 50 µm, with red and green channel being represented. **b** Fluorescence intensity of OG-FAM and TAMRA was measured using wide-field images in ImageJ and the ratio is plotted against the time of incubation starting after 7 h when microcolony formation occurred. Error bars represent standard error measured for different areas of the images, where *n* = 6. To view the movie, see Supplementary Movie 1.

Once the microcolonies started to form after 10 h, the colour around the microcolonies changed to red indicating an acidification during the microcolony formation. Red streaks could be observed after 13 h coming from the microcolonies and being taken away by the flow (Fig. 4a). Subsequently, the colour continued changing towards red until the whole biofilm was acidified. This observation was confirmed by analysing the fluorescence intensity of the green channel (pH-sensitive dyes) and red channel (reference dye) using ImageJ (Fig. 4b). Over the first hours when microcolonies started to form, the fluorescence intensity ratio of OG-FAM and TAMRA increased slightly from 1.5 to 1.6, indicating a pH increase. The fluorescence intensity around the microcolonies starts to decrease after 10 h before the intensity of the medium declines. After 16 h of biofilm growth, the fluorescence intensity ratio around the microcolonies as well as within the medium reaches 1.25, indicating an acidification of the whole biofilm. A video of the time-lapse imaging can be found in supplementary (Supplementary Movie 1). The experiment was terminated after 22 h to prevent blocking of the BioFlux channel.

### Single microcolonies of a PAO1-N biofilm show pH variation from a more acidic environment within the core to a more neutral pH on the outside surface

**Fig. 5:**
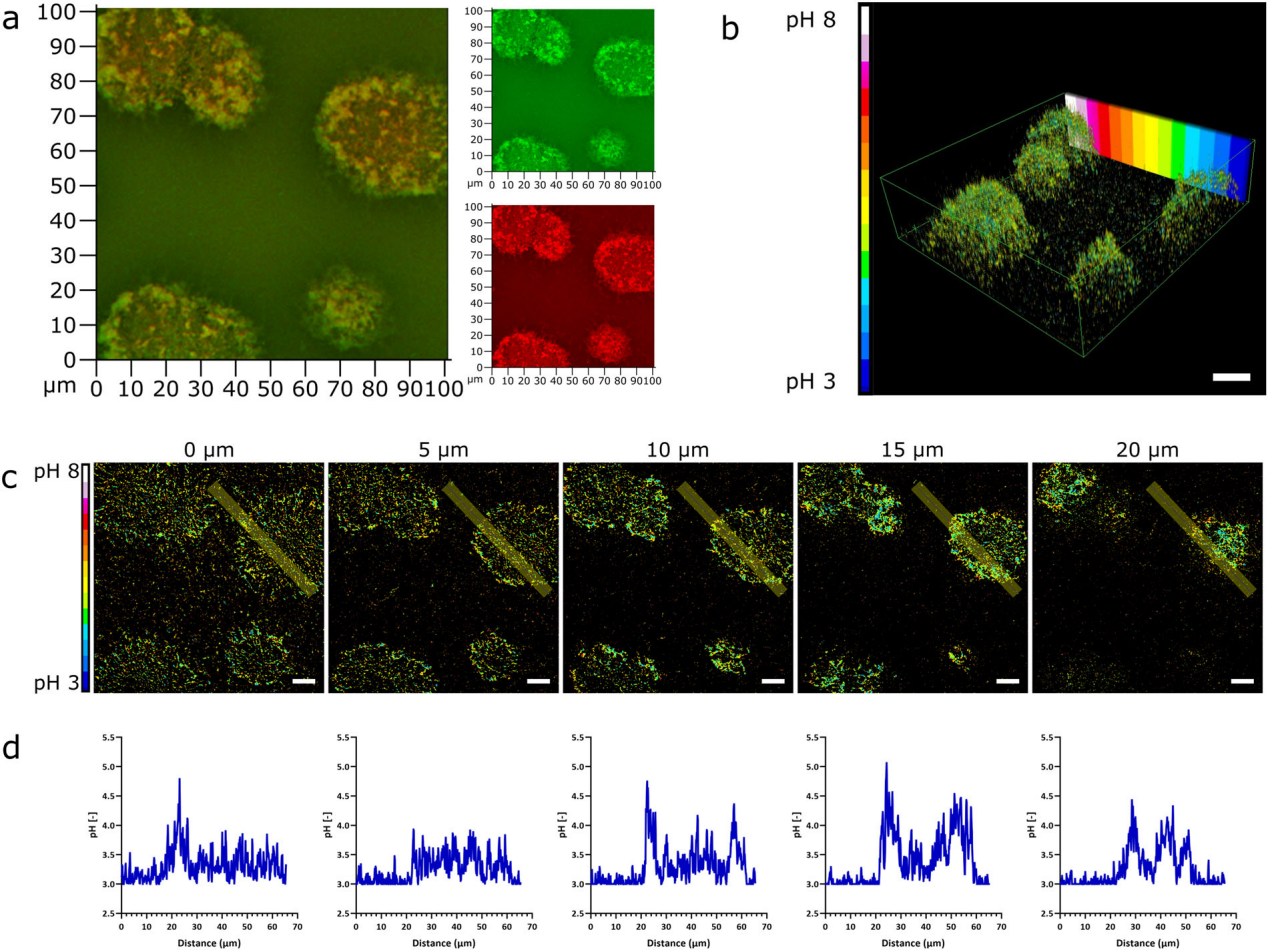
*P. aeruginosa* microcolonies in flow biofilms have an acidic centre. **a** The confocal 3D *Z*-stack image of a representative single microcolony from a *P. aeruginosa* biofilm grown in a BioFlux flow cell system with 5 mg mL^−1^ neutral charged pH-sensitive polyacrylamide nanosensors (fluorescence overlay, OG-FAM fluorescence in green, TAMRA fluorescence in red). The pH variation was observed after biofilm growth for 22 h. MatLab and ImageJ analysis of confocal images shows a lower pH of 3.5–4.0 within the core of microcolonies and a higher pH of 5.5–6.0 at the outer parts. Images represent 3D rendering of the biofilm *Z*-stack (**b**) and individual pH maps at the bottom of the biofilm (0 µm) and 5, 10, 15 and 20 µm from the bottom into the biofilm (**c**). Plot profiles of a straight line highlighted within the pH maps were created using ImageJ (**d**). Scale bars represent 10 µm.

CLSM was used to look at the pH variation of single microcolonies of a *P. aeruginosa* PAO1-N biofilm grown within a BioFlux flow cell. The biofilm was imaged in 1 µm steps up to 25 µm. The 3D *Z*-stack shows a colour change from red within the core of the microcolonies towards yellow at the outer parts (Fig. 5a). After calibration of the nanosensors using pH buffers ranging from pH 3 to 8 (Supplementary Fig. 4), pH maps and a 3D rendering of the *Z*-stack of the biofilm were generated using the software MatLab^68^. The values of the pH maps correlate with the observations from the 3D *Z*-stack showing a low pH within the microcolonies (~pH 3.5–4.5) and a more neutral pH towards the outside (pH 5.5–6.0) (Fig. 5b–d). A video of the 3D rendering can be found in Supplementary Movie 2.

### The addition of glucose drastically reduces the pH of the medium when added to starved planktonic cells and biofilms of *S. mutans*

Having optimised the inclusion of positive pH-sensitive polyacrylamide nanosensors in *P. aeruginosa* biofilms under both static and flow conditions, we applied our optical nanosenors to the analysis of our second bacterial model with the aim to translate our fundamental observations to an applied scenario. Acidification of the environment by the oral pathogen *S. mutans* linked to the presence of its preferred sugars, would provide a valuable method to assess potential treatments for dental caries. Bearing this in mind, the response of starved planktonic *S. mutans* to the introduction of fermentable carbon sources (glucose and sucrose) versus a non-fermentable carbon source (xylose and xylitol) was analysed as well as the addition of glucose or water to *S. mutans* biofilms, with the aim of detecting any pH changes in the medium as an indirect measurement of the fermentation of a carbon source.

**Fig. 6:**
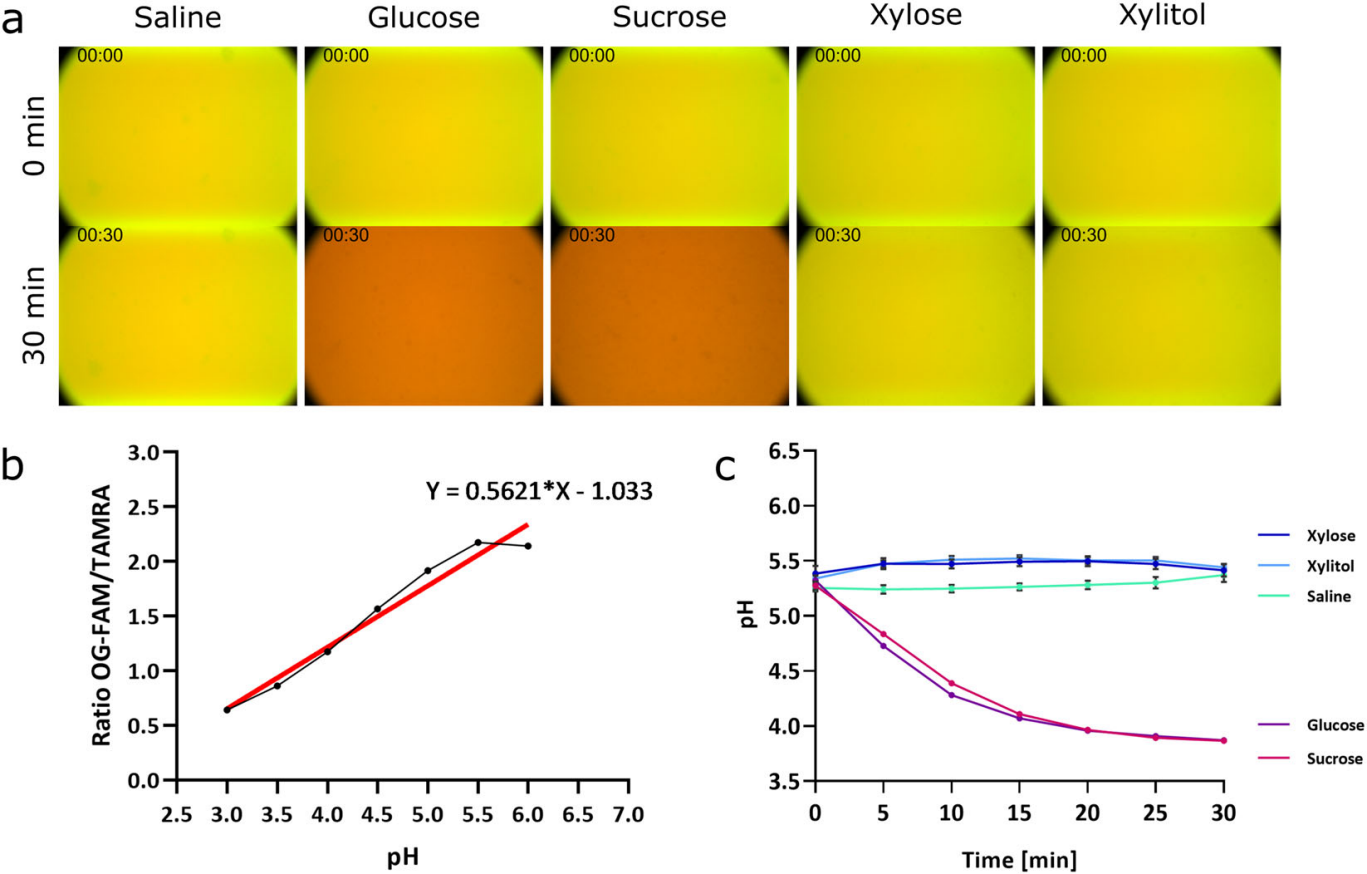
Fermentation of glucose and sucrose, but not xylose, xylitol, or saline by *S. mutans* visualised with pH-sensitive nanosensors. Positively charged pH-sensitive nanosensors at a concentration of 1 mg mL^−1^ were added to planktonic *S. mutans* NCTC 10449 grown overnight and normalised to an OD_600_ of 1. Time-lapse images were taken with a Nikon widefield microscope. **a** Representative fluorescence images of cells with nanosensors in saline before the addition of either saline, glucose, sucrose, xylose or xylitol (top panel) to a final concentration of 1% or after 30 min (bottom panel). An overlay image of the fluorescence channels for green (OG-FAM) and red (TAMRA) is shown. **b** pH calibration of the nanosensors using the ratio between OG-FAM and TAMRA calculated from the fluorescence intensities. Linear regression was calculated and used for pH determination of samples. **c** The pH of each condition at the indicated timepoints was calculated and plotted against the time of addition. Error bars represent standard error measured for different images, where *n* = 9.

Fluorescence microscopy revealed that looking at planktonic cells, the control with saline remained unchanged throughout the experiment. This was also the case following the introduction of the non-fermentable xylose and xylitol, which resulted in no significant change in the pH during the 30 min period. In contrast, during the same time period, the addition of both glucose and sucrose to the starved cells led to a drastic reduction in pH from ~pH 5.3 to ~pH 3.8 and ~pH 5.2 to ~pH 3.9, respectively (Fig. 6).

**Fig. 7:**
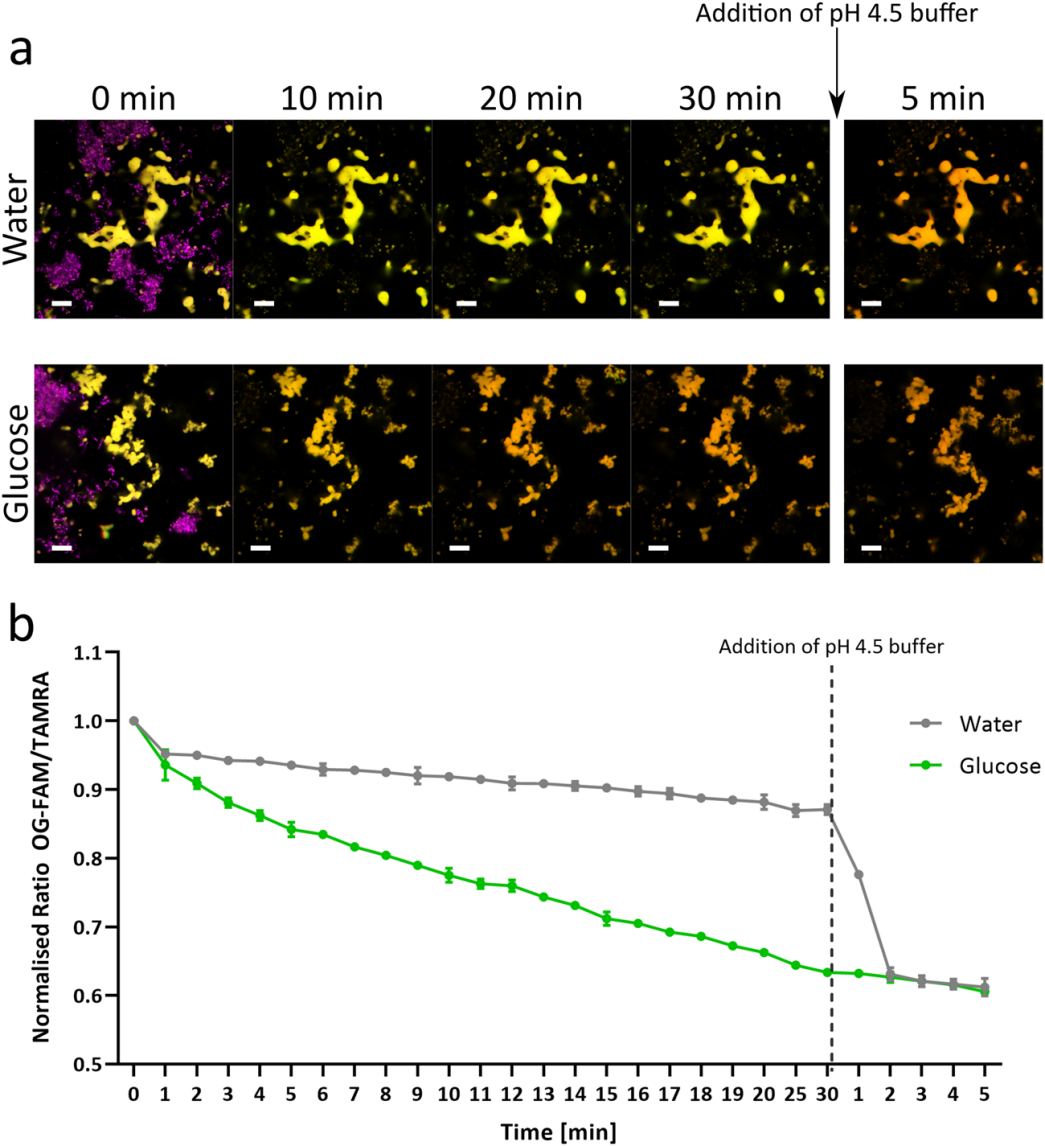
Reduction in fluorescence intensity is a result of glucose metabolism over time in biofilms of *S. mutans*. Biofilms of *S. mutans* NCTC 10832 were initially imaged after 48 h of static growth before either water or 1% glucose was added to the biofilm. Further images were taken every minute for 20 min, and then at 25 and 30 min. After 30 min, a 4.5 pH buffer was added and further images were taken every minute for 5 min. **a** 3D top view fluorescent microscope image of the fluorescence intensity changes over time. Green (OG-FAM), red (TAMRA) fluorescence with time point 0 min including DAPI-stained *S. mutans* cells shown in magenta. Scale bar represents 10 µm. **b** Fluorescence intensity of OG-FAM and TAMRA was measured using wide-field images in ImageJ and the normalised ratio is plotted against the time of incubation. Error bars represent standard error measured for different biofilm replicates, where *n* = 3.

In addition, the response of an *S. mutans* biofilm to a glucose challenge was examined by introducing 1% glucose into the medium hydrating the biofilm. Measuring fluorescence intensity using CLSM revealed a reduction in the fluorescence intensity ratio when the *S. mutans* biofilm was challenged with 1% glucose (Fig. 7), while there was only a slight reduction in the fluorescence intensity ratio when distilled water is added. These changes in fluorescence intensity can be visualised in the time-lapse panel as the colour changes from yellow to orange, caused by the reduction in fluorescence from the pH-sensitive fluorophores, OG and FAM. The decrease in fluorescence intensity with the addition of a 4.5 pH buffer furthermore confirms that the nanosensors are responsive to induced pH changes.

## Discussion

This study has shown that pH-sensitive polyacrylamide nanosensors penetrate biofilms and report on pH modulation for both our chosen model bacteria, *P. aeruginosa* and *S. mutans*. Once nanosensors have been integrated into biofilms, they are capable of real-time pH quantification in static model systems at high temporal, spatial and measurement resolution that permits determination of (i) a pH gradient across the biofilm thickness and also (ii) an acidic core to microcolonies. These studies also include time-lapse imaging that reveals acidification downstream of microcolonies in a flow biofilm model of *P. aeruginosa*. Moreover, in static models, nanosensors with a positive charge supported the formation of thicker *P. aeruginosa* biofilms. Finally, detection of the previously established fermentation of preferred sugars by *S. mutans* has the potential to provide the opportunity to develop a tool to test and understand treatments intended to reduce the progression of dental caries.

The selection of appropriate nanosensors, especially with regard to their charge, is crucial for the analysis of microbial biofilms. The nanosensors chosen for this work are well suited to analyse pH changes over time using time-lapse imaging as well as to look at pH variation within single microcolonies using CLSM. The biofilm thickness promoting effect of positively charged polyacrylamide nanosensors upon static PAO1-N biofilms was independent of the incorporation of fluorophores and conferred an enhanced robustness to the biofilms. Penetration of biofilms by nanoparticles usually occurs by diffusion, with particles interacting with the bacteria and the biofilm matrix components^69–72^. Due to the negative surface charge of PAO1-N, it is likely that the positively charged nanosensors are interacting directly with the bacteria, and this was supported by the super-resolution microscopy (Supplementary Fig. 2). This interaction could facilitate the bacteria to attach to each other and form a denser biofilm as seen here. The nanosensors would in this case function as ‘glue’, sticking the cells together and enable biofilm development. The nanosensors are thus well placed to report on the pH in the vicinity of the bacterial cells. When neutral nanosensors or negatively charged particles are used, this interaction would be predicted to be reduced or even reversed most likely due to the same charge of the bacteria and nanoparticles leading to repulsion instead^53,73^. The enhanced biofilm formation was not observed when biofilms were grown in the BioFlux flow cell system. Due to the constant flow of medium, it could be postulated that the bacteria are less influenced by the nanosensors and therefore their adherence to each other is not modulated.

A systematic investigation is underway to decipher how the nanosensors interact with bacteria in biofilms, taking advantage of strain variation and defined mutations to determine whether there are interactions with matrix components (e.g. polysaccharides/eDNA).

When positively charged nanosensors were added to biofilms that were initially grown without nanosensors, the biofilm growth structure was disrupted. This behaviour was not seen when positively charged nanoparticles were present throughout biofilm formation. One hypothesis that could be drawn from this is that biofilms initially grown with positive nanoparticles were more robust. In line with this, Li et al. reported that positively charged block copolymer nanoparticles dispersed established biofilms of multidrug-resistant Gram-positive bacteria^74^. The situation appears to differ in flow cell biofilms since both neutral and positively charged nanosensors were able to penetrate an already established biofilm that had been grown for 12 h. Even after changing the flow cell back to a medium without nanosensors, the nanosensors were still detectable and incorporated within the biofilm. Distribution of both types of nanosensors was extensive in the flow, with the neutral nanosensors marginally more evident. This has the practical application for future investigation as it indicates that the nanosensors can be added at later timepoints for pH analysis if needed.

Being able to increase the thickness of biofilms has beneficial implications. For example, the positively charged nanosensors could be employed in future studies to elucidate the mechanistic steps of biofilm formation by combining them with mutants defective in defined aspects of biofilm formation. On the other hand, neutral nanosensors could be the better choice to study biofilms that closely mimic the natural physiological or environmental conditions, as no structural change in biofilm formation behaviour was observed when they were applied to our models.

This study has shown, that pH-sensitive polyacrylamide nanosensors can be used to visualise pH changes over time during biofilm formation of PAO1-N within a BioFlux flow cell system at a microcolony level. At the beginning of the biofilm formation, the pH increases very slightly as the bacteria start to adapt to the environment. After 10 h, acidification of the biofilm was observed during microcolony formation and subsequently while the biofilm continued to form. The acidic streaks that were released by the microcolonies could be exoproducts from the bacteria, produced during metabolism^53^. Another possibility is that acidic molecules are released by the cells to help form the biofilms. These molecules could, for example, be eDNA or exopolysaccharides (EPS). It could also be that the cells are actively lowering the pH when biofilm formation is initiated, to facilitate the process, for example to enable better attachment or to increase the turn-over and release of QS molecules like AHLs^44,75^. Further studies will address the pH gradients across the entire flow channel and under the influence of different flow velocities to help us understand the environments to which bacteria in biofilms are exposed.

In addition to the pH changes observed over time within the whole biofilm, pH variation was also detected within single microcolonies, showing a more acidic environment within the core of the microcolonies and a neutral pH at the outside edge of the colonies. The acidic cores could result from the production and release of acidic metabolites during growth and biofilm formation. Furthermore, oxygen will likely be depleted within the core and no fresh oxygen would reach the inner part of the microcolonies resulting in fermentation processes of the cells, which would then lead to the production of acidic products, reducing the pH^38^. Previously, slightly higher pH gradients (pH 5–7) were reported for biofilms of *P. fluorescens* and *E. coli*^53,60^. However, these biofilms were grown under static conditions as opposed to flow cell systems, with different organisms and growth media. Other studies reported pH values measured in the centre of microcolonies of as low as 4.1 (ref. ^7^).

To provide a translational benefit of the pH-sensitive nanosensors, they were dispersed within the medium of starved planktonic *S. mutans*, to measure changes in fluorescence intensity and calculate the resultant external pH changes. By utilising the well-studied biological system of *S. mutans*, where carbohydrate supplementation can induce a pH response, the effectiveness of the pH-sensitive polyacrylamide nanosensors as a real-time tool to accurately determine pH changes in extracellular media was illustrated. Oral bacteria including *S. mutans* must adapt to the shifting availability of carbohydrates caused by changes in host behaviours. These can be brought on through the fluctuation of diets, as well as through host secretions of glycoproteins and those carbohydrates produced by the oral microbiome itself^76^. *S. mutans* is therefore versatile in the carbohydrates it can utilise^77^. With the addition of simple mono- and disaccharides such as glucose or sucrose, a reduction in the pH of starved planktonic cultures as well as within biofilms of *S. mutans* was observed as expected, as *S. mutans* is capable of metabolising both glucose and sucrose via glycolysis to produce pyruvate^27^. The resultant pyruvate is metabolised to form l-lactate which would be secreted in the form of lactic acid, leading to the reduction of the pH of the medium^78^. However, with the addition of xylose or its derivative, xylitol, as would be predicted from the literature, the external pH was unchanged throughout the experiment. This was a result of both xylose and xylitol being non-fermentative by *S. mutans*^79^. Xylose is initially taken up and reduced to xylitol, which is then phosphorylated to xylitol-5-phosphate (X5P). *S. mutans* is unable to metabolise X5P further, and as a result X5P is accumulated intracellularly^80^. This accumulation of X5P has been attributed to the inhibition of glycolytic enzymes, leading to the repression of acid production^80,81^. However, Takahashi and Washio^33^ were able to show that the presence of X5P had no effect on acid production when supragingival plaques were rinsed with glucose after an initial application of xylitol. The implication that xylitol is simply a non-fermentative sugar alcohol rather than an inhibitor is supported by our observations.

A practical application of our nanosensor system could be envisioned for testing oral hygiene products or sweetener alternatives in soft drink production, in order to determine the impact on pH production. The nanosensors would provide flexibility, as a screening tool to measure pH changes in high throughput fluorescence assays. Alternatively, the nanosensors could be used as a more focused tool, to track changes in the pH microenvironment of established oral biofilms over time, to map 3D pH microenvironments as highlighted by our analysis in the flow cell.

While there are some limitations to the application of the nanosensors including the need to synthesise them, the variability in their interaction with bacteria in certain scenarios, and the need to calibrate their accuracy within each experiment, they offer sensitive, high resolution and dynamic detection.

Future work will also explore the molecular mechanisms underpinning the detected acidification of the biofilms and microcolony centres. For example, different fluorophores could be used to stain eDNA or EPS components to investigate whether either correlates with the acidic streaks observed. Furthermore, adding or changing dyes in the nanosensors could enable more powerful tools to be developed for monitoring broader environmental microniche changes within biofilms in real time and at high resolution.

## Methods

### Materials

Oregon Green® 488 carboxylic acid (OG), 5-(and-6)-carboxyfluorescein (FAM) and 5-(and-6)-carboxytetramethylrhodamine (TAMRA) were obtained from Invitrogen^TM^, USA. Acrylamide (≥99%), *N*,*N*′-methylenebis(acrylamide) (bisacrylamide, 99%), dioctylsulfosuccinate sodium (AOT), ammonium persulfate (APS, ≥98%), 3-acrylamidopropyltrimethyl ammonium hydrochloride (ACTA, 75 wt% in H_2_O), polyoxyethylene(4)lauryl ether (Brij L4®), sodium tetraborate decahydrate (≥99.5%) and *N*,*N*,*N*,*N*-tetramethyl-ethylenediamine (TEMED, 99%) were purchased from Sigma Aldrich, US. *N*-(3-aminopropyl) methacrylamide hydrochloride (APMA, >98%) was obtained from Polysciences Inc., Germany. Hexane, ethanol absolute (99.5%) and phosphate buffer saline (PBS) were obtained from Fisher Scientific, UK. Unless otherwise mentioned, all the chemicals that used throughout this study were of analytical grade.

### Minimal medium (M9)

Minimal medium (M9) was prepared by using 200 mL of 5× M9 salts (Sigma Aldrich), 2 mL MgSO_4_ (1 M), 100 µL CaCl_2_ (1 M) and 40 mL succinate (0.5 M) in a total of 1 L.

### Bacterial strains and growth culture

The bacterial strains used in this study were *P. aeruginosa* PAO1-N wild type and *S. mutans* NCTC 10449 and 10382 (ref. ^82^). Lysogeny broth (LB)^83^ was used as standard growth medium (liquid or solid as agar plate) for PAO1-N, Brain Heart Infusion (BHI) for NCTC 10449 or Todd Hewitt (TH) for NCTC 10832. *P. aeruginosa* strains were grown at 37 °C for 16–20 h; *S. mutans* strains were grown at 37 °C for 48 h in 5% CO_2_. Liquid cultures were prepared in 5 mL liquid media, inoculated with a single bacterial colony and shaken at 200 r.p.m. (PAO1-N) or incubated in a static incubator (*S. mutans*) overnight. Strains were stored for the longer term as glycerol cultures which were made by using 500 µL overnight culture with 500 µL sterile 50% glycerol and stored at 80 °C.

### Static biofilm growth of PAO1-N

Overnight cultures of *P. aeruginosa* grown in LB were used to spin down 1 mL of cells in a fresh Eppendorf tube at 1000 × *g* for 1 min. The pellet was re-suspended in 1 mL phosphate buffer saline (PBS), spun down again at 1000 × *g* and re-suspended in 1 mL of minimal medium (M9 succinate). The OD_600_ was taken and the cells normalised to 0.5 OD_600_ in 1 mL of selected medium. Nanosensors were suspended at 1.5 mg mL^−1^ in selected medium. Polyacrylamide nanosensor suspensions were filter sterilised using 0.22 µm PES filters and 500 µL of medium with nanosensors were combined with 175 µL medium and 75 µL normalised cells to obtain a final concentration of 1 mg mL^−1^ nanosensors and a starting OD_600_ of 0.05. For approaches without nanosensors, another 175 µL medium was added instead of medium with nanosensors. To each well of the eight-well chamber (Ibidi, glass bottom), 300 µL of the suspension was added before the chamber was placed in a box wrapped in aluminium foil and placed in a static incubator at 37 °C. After an incubation of 48 h, the media was carefully removed and replaced by fresh medium containing 2.5 µg mL^−1^ CellMask™ Deep Red plasma membrane stain (Thermo Fisher Scientific).

### Flow cell biofilm of PAO1-N growth using BioFlux

Overnight cultures of *P. aeruginosa* grown in LB were used to set up liquid cultures in 5 mL LB using 100 µL of the overnight culture. The cultures were grown to 0.4–0.8 OD_600_ and normalised to an 0.05 OD_600_ in 1 mL of minimal medium (M9 succinate). Nanosensors were suspended at 5 mg mL^−1^ in selected medium and filter sterilised using 0.22 µm PES filters. The BioFlux flow cell (BioFlux 200 48-well low shear plate, 0–20 dynes cm^−^^2^) was prepared according to the manufacturer’s instructions (Fluxion Biosciences, BioFlux System). For the seeding of the cells, 50 µL of the normalised cells was used with a seeding time of 45–60 min. After the seeding, 1 mL of the prepared medium with or without nanosensors was used as flow medium. The flow was set to 0.25 dyn cm^−2^ and the system was run for 20–24 h. Time-lapse imaging was applied using a Nikon widefield microscope with brightfield and fluorescence channels (OG-FAM excitation = 460 nm, TAMRA excitation = 550 nm). Images were taken every 15 min for 16 h.^84^

### Sugar challenge with planktonic NCTC 10449 cells

An overnight culture of *S. mutans* NCTC 10449 in BHI was spun down and re-suspended in PBS for 30 min as a starvation step. The cells were spun down again and re-suspended in saline before being mixed with positively charged nanosensors in saline to obtain an OD_600_ of 1 and a nanosensor concentration of 1 mg mL^−1^. The suspension was aliquoted into a *Greiner* 24-well, PS, flat glass bottom, black walled microplate using 400 µm per well. Imaging was performed using widefield fluorescence microscopy with the ×40/0.6 lens (×40 objective with ×1.5 additional magnification). An image was taken (brightfield, OG-FAM excitation = 460 nm, TAMRA excitation = 550 nm) for time point 0 min before 20 µL of either glucose, sucrose, xylose, xylitol (all 1% final conc.) or saline was added to their respective wells. Images were taken every 5 min for 30 min. Additional controls with glucose, sucrose, xylose, xylitol and saline solution added to 1 mg mL^−1^ positively charged nanosensors in saline were also imaged to confirm the absence of any pH change brought on by the solutions themselves.

The calibration was performed using a Greiner 24-well microplate where pH buffers from pH 8 to pH 2.5 were mixed with positively charged polyacrylamide nanosensors for a final concentration of 1 mg mL^−1^. Images were taken from each pH using the same exposure settings as the experiment. The fluorescence intensity ratio from each pH was plotted and the linear regression calculated to determine the pH values from the fluorescence intensities generated during the experiment.

### Glucose challenge with biofilms of NCTC 10832

*S. mutans* strain NCTC 10832 was initially streaked out from a −80 °C stock onto TH agar and incubated at 37 °C, 5% CO_2_ for 48 h before being stored at 4 °C for future use. Overnight cultures were made using 5 mL TH media and an individual colony picked with a sterile 10 µm loop from the streaked plate. The culture was incubated overnight at 37 °C in a static incubator and 1 mL of NCTC 10832 inoculum was centrifuged at maximum speed for 1 min. The pellet was washed in 1 mL PBS and re-centrifuged at maximum speed for 1 min before the pellet was re-suspended in TH + 1% sucrose. The biofilms were set up with a cell suspension of NCTC 10832 at OD_600_ = 0.05 mixed with 1 mg mL^−1^ positively charged polyacrylamide nanosensors in TH media + 1% sucrose and incubated for 48 h, 37 °C, 5% CO_2_. After 48 h, the media was replaced with TH media alone and incubated for a further 24 h. The imaging was performed using CLSM with the ×63/1.46 oil objective lens. The media was removed and replaced with saline (0.9% NaCl) while DAPI (1 mg mL^−1^) was added to detect cells during the process. A plane of interest was found and either glucose (1%) or water was added. Images were taken every minute for 20 min, and then 25 and 30 min. After 30 min, a 4.5 pH buffer was added to each well and an image was taken every minute for 5 min. The biofilms were imaged using a Zeiss confocal laser scanning microscope and the appropriate excitation settings for the fluorescence channels (Laser: OG-FAM = 488 nm, TAMRA = 555 nm; 2% laser power; emission: OG-FAM = 496 nm, TAMRA = 560 nm). Duplicate images were taken at 0 and 5 min post pH treatment with the addition of DAPI (laser = 405 nm, emission = 420 nm) to detect the cells.

### Nanosensor preparation

A 50 mM (pH 9.5) Sodium tetraborate decahydrate buffer in water was prepared and used to make a 0.002 mmol APMA solution. For each fluorophore, 1 mg was weighed out in separate vials and suspended in 200 µL of the APMA solution. The mixtures were sonicated for 5 min before being incubated on an oscillator and room temperature for 24 h in the dark. The dyes were stored at −20 °C for further use.

For the synthesis of the nanosensors, 1.59 g AOT and 3.08 g Brij L4 were weighed out, mixed in a 250 mL round bottom flask and deoxygenated for 15–20 min using argon while being stirred. A 500 mL round bottom flask was used to deoxygenate 100 mL hexane for 30 min using argon before 42 mL of the deoxygenated hexane was added to the AOT and Brij solution. The flask was sealed with a stopper and a balloon under an argon atmosphere with continuous stirring. For the synthesis of neutral polyacrylamide nanosensors, 540 mg acrylamide and 160 mg bisacrylamide were dissolved in 1.5 mL deionised water, whereas 513 mg acrylamide, 152 mg bisacrylamide and 119 µL ACTA were used the preparation of positively charged polyacrylamide nanosensors. The APMA-fluorophore conjugates were added to the acrylamide solution using 15 µL OG-APMA, 15 µL FAM-APMA and 60 µL TAMRA-APMA. The mixture was deoxygenated and added to the flask containing the surfactants and hexane using a syringe. After 10 min, 30 µL of APS (10% w/v) and 15 µL TEMED were added. The flask was deoxygenated again, sealed, wrapped in aluminium foil and incubated on the stirrer for 2 h. After the incubation, the hexane was removed using a rotary evaporator at 30 °C and 30 mL of ethanol (100%) was added. The mixture was transferred to a 50 mL falcon tube and spun down at 3800 × *g* for 3 min. The supernatant was discarded, and the pellet re-suspended in 30 mL of ethanol (90% in water). The mixture was spun down again at 3800 × *g* for 3 min and washed twice in 30 mL ethanol (100%) and then suspended in 10 mL of ethanol (100%). The mixture was transferred into a clean 250 mL flask and the ethanol was removed using the rotary evaporator at 30 °C before the nanosensors were collected and stored at −20 °C in the dark until further use.

### Nanosensor calibration and pH calculation using Matlab

Calibration was undertaken in conditions matching each experiment, for each batch and following long term storage. Buffer solutions with a pH ranging from 3.0 to 8.0 in 1.0 steps were prepared using 0.2 M sodium phosphate (Na_2_HPO_4_) and 0.1 M citric acid (C_6_H_8_O_7_) as indicated in Supplementary Table 1. The pH was adjusted using 0.2 M sodium phosphate and 0.1 M citric acid.

For the pH calibration of the nanosensors in a BioFlux experiment, 10 mg mL^−1^ polyacrylamide were prepared in deionised water and mixed 1:1 with each of the pH buffers to get a concentration of 5 mg mL^−1^. To enable the calculation of a calibration curve, 500 µL of each solution were added to a BioFlux plate and was run for 2 min with a flow rate of 0.25 dyn cm^−^^2^. Images were taken for each buffer sample using a Zeiss CLSM (OG-FAM = 488 nm, TAMRA = 555 nm). The software MatLab was used to calculate the calibration curve and subsequently to determine the pH of the actual biofilm images taken with the Zeiss CLSM.

### Zeta potential and size measurement of nanosensors

For the determination of the zeta potential, 1 mg mL^−1^ nanosensors were prepared in PBS (10%) and transferred into a disposable folded capillary cell using a 2 mL syringe. For the size determination of polyacrylamide nanosensors, 1 mg mL^−1^ nanosensors were prepared in PBS (10%) and transferred into a cell disposable cuvette. Both the zeta potential and the size were measured using the Zetasizer.

### Widefield microscopy

Time-lapse imaging and planktonic sugar challenge were performed using a Nikon eclipse Ti2-U widefield microscope fitted with a Nikon S Plan Fluor ELWD 20x/0.45, Plan Fluor ×10/0.30 or CFI60 ×40x/0.6 objective lens. Images were captured by using a CoolSNAP™ MYO CCD Camera connected to Nikon software and analysed using ImageJ.

### Confocal laser scanning microscopy

To generate more detailed images, a Zeiss LSM 700 compact confocal laser scanning microscope was used with a Zeiss alpha-Plan-Apochromat ×63/1.46 oil objective lens. The biofilms were imaged using a Zeiss confocal laser scanning microscope with the ×63/1.46 oil objective lens and the appropriate excitation settings for the fluorescence channels (laser: DAPI = 405 nm, CellMask = 639 nm, OG-FAM = 488 nm, TAMRA = 555 nm; 2% laser power; emission filters: DAPI = 420 nm, CellMask = SP 640 nm, OG-FAM = LP 555 nm, TAMRA = SP 640 nm). Images were recorded and 3D rendered using ZEN software and analysed using Zen blue software.

### Reporting summary

Further information on research design is available in the Nature Research Reporting Summary linked to this article.

## Supplementary information

Supplementary Information

Supplementary movie 1

Supplementary movie 2

Reporting Summary

## Data Availability

All data generated or analysed during this study are included in this published article (and its Supplementary Information files).
