# Supplementary material for: Fluorescent nanosensors reveal dynamic pH gradients during biofilm formation
Source: NPJ Biofilms Microbiomes. 2021 Jun 17;7:50. doi: 10.1038/s41522-021-00221-8 (PMC8211749; doi:10.1038/s41522-021-00221-8)
Supplement: Supplementary file 1 — Supplementary Information [file 41522_2021_221_MOESM1_ESM.pdf]

Supplementary Figure 1. No growth inhibition by nanosensors at concentrations below 25 mg mL^{-1}.

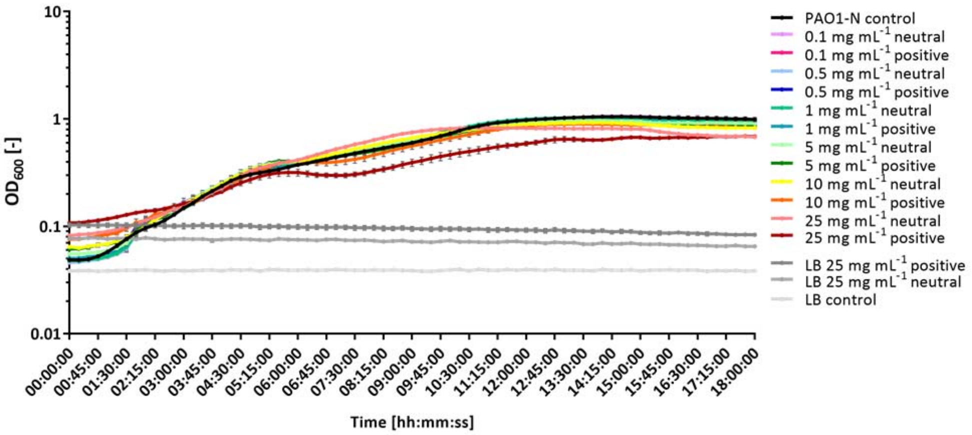

PAO1-N was inoculated into a 96-well plate at an OD_{600} of 0.05 in LB media containing 0.1 to 25 mg mL^{-1} of neutral or positive nanosensors and incubated for 24 h at 37°C, during which the OD_{600} was measured automatically every 15 minutes and is shown on a log scale against time. No path length correction was applied. Error bars represent standard error, where n = 3x3

Supplementary Figure 2. Representative images of PAO1-N show some leftover positive nanosensors stuck between cells after being washed but no fluorescence inside the cells.

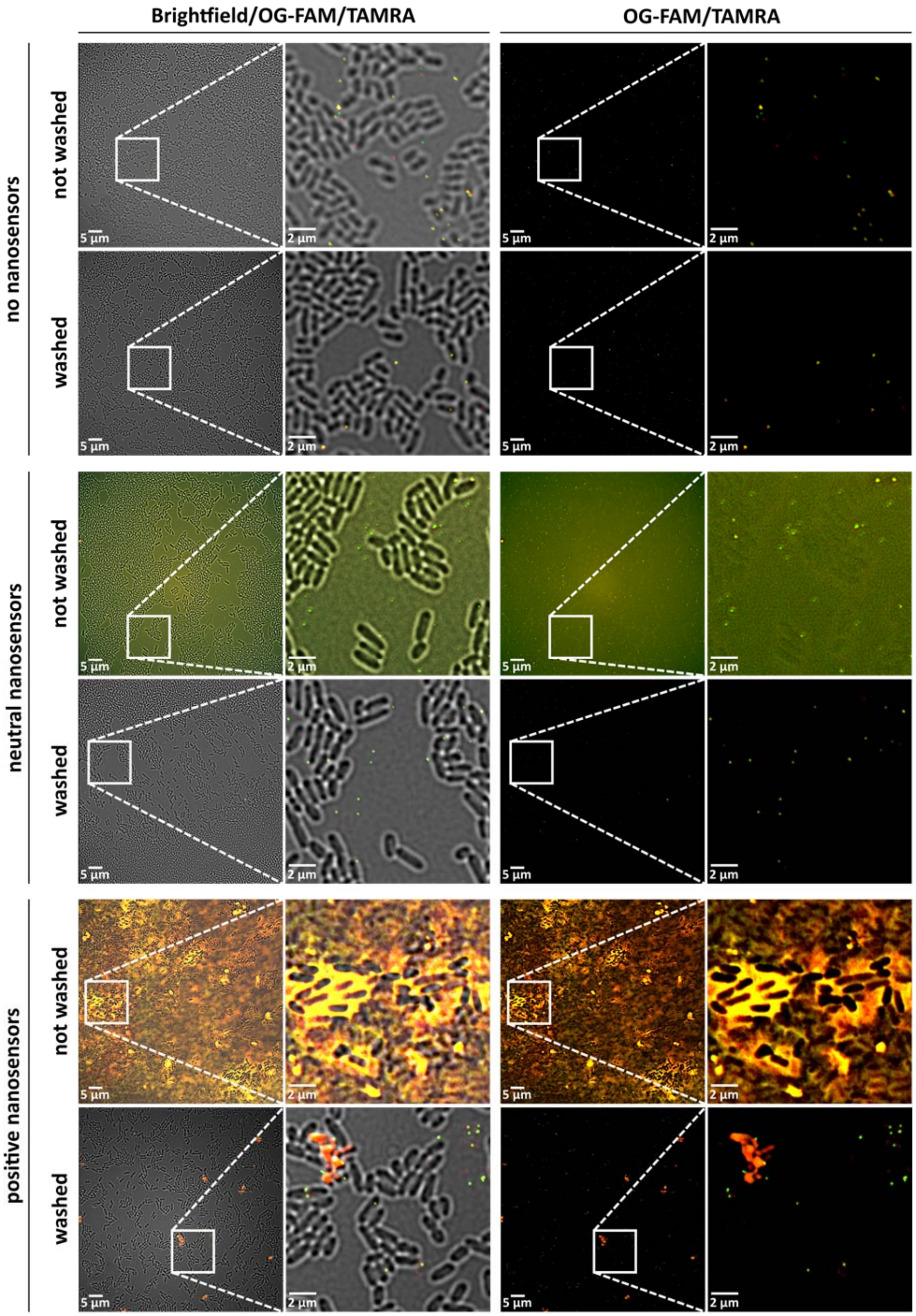

PAO1-N overnight cultures were incubated in LB with or without 1 mg mL^{-1} positive or neutral nanosensors at 37°C. For all three overnight cultures, an aliquot was pelleted by centrifugation and

resuspended in fresh LB broth. The wash step was performed twice to remove all extracellular nanosensors, that are evident as a coloured background haze in the unwashed samples. Washed or unwashed cells were imaged using a Zeiss Elyra PS.1 with structured illumination microscopy and a 63x objective. Four-coloured (green, red, blue, far-red) fluorescent TetraSpeck™ Microspheres were used for image processing.

Supplementary Figure 3. Enhanced *P. aeruginosa* biofilm formation as a result of positively charged nanosensors is concentration dependent.

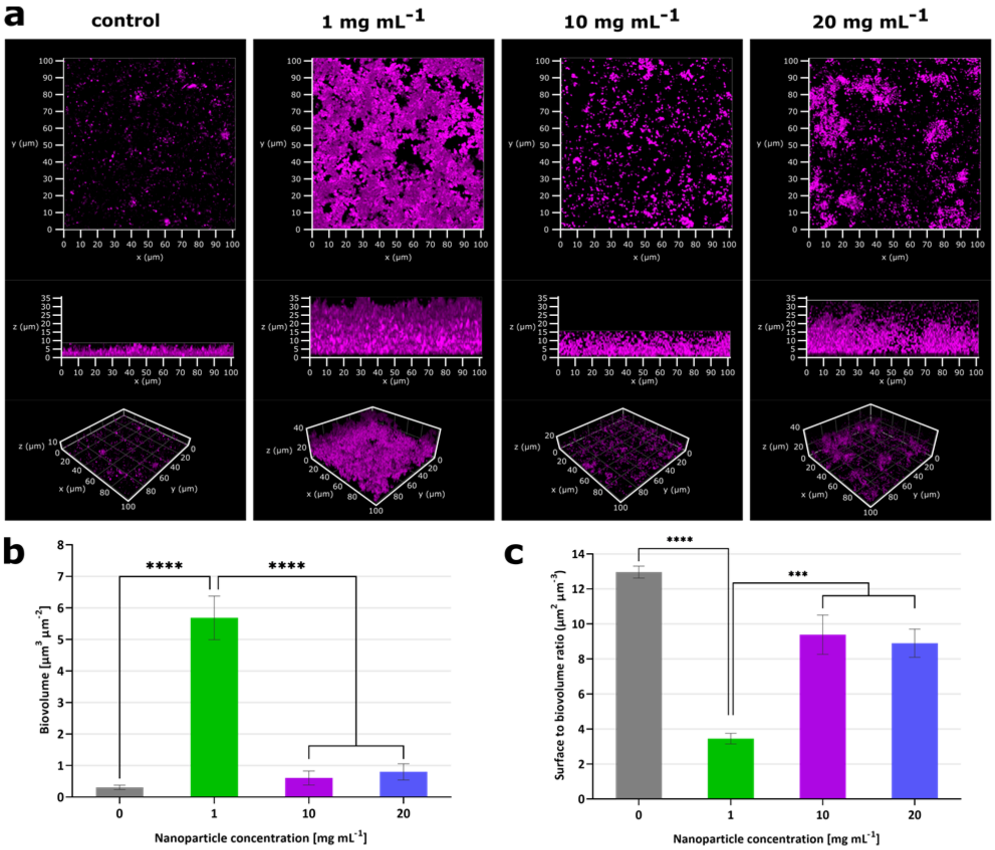

(**a**) Representative confocal images of *P. aeruginosa* PAO1-N stained with CellMask™ grown statically for 48 h without the addition of nanoparticles (left panel) or with positive polyacrylamide nanosensors at 1 mg mL1 (left central panel), 10 mg mL1 (right central panel) or 20 mg mL1 (right panel). The top row is a 3D view from the top of the biofilm, the bottom row shows a 3D view from

the front of the biofilm. Quantitative Comstat analysis of the biofilm images were performed using ImageJ. Graphs represent (**b**) biomass and (**c**) surface to biovolume ratio. Error bars represent standard deviation measured for different biofilm images, where n=10 (control), n=8 (1 mg mL^{1}), n=12 (10 mg mL^{1}) and n=13 (20 mg mL^{1}), with $p<0.0001$ represented by **** and $p<0.001$ represented by *** (ordinary one-way ANOVA). The same number of replicas were visually inspected.

Supplementary Figure 4. Calibration curve of nanosensors within a BioFlux plate measured using CLSM and automatically calculated using MatLab.

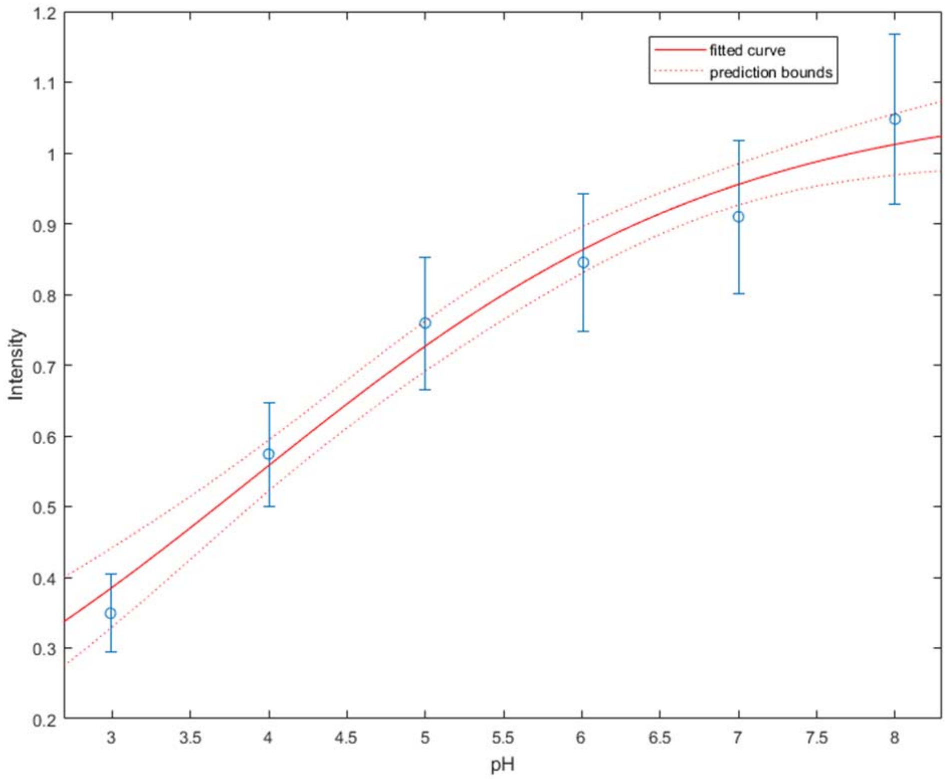

Nanosensors were suspended in pH buffers ranging from pH 3 to pH 8 at a concentration of 1 mg mL^{-1}. Images of nanosensor solutions were taken in duplicates for each pH using a CLSM under the same conditions as the corresponding experiment and analysed using MATLAB.

Supplementary Table 1. pH buffer preparation using 0.2 M Sodium phosphate (Na_2HPO_4) and 0.1 M Citric acid ($C_6H_8O_7$).

pH	Na_2HPO_4 [mL]	$C_6H_8O_7$ [mL]	H_2O [mL]
3.0	2.04	7.96	10
4.0	3.86	6.14	10
5.0	5.14	4.86	10
6.0	6.42	3.58	10
7.0	8.72	1.3	9.98
8.0	9.765	0.24	9.99

Na_2HPO_4 0.2 M Sodium phosphate, $C_6H_8O_7$ 0.1 M Citric acid, H_2O water

Supplementary Movie 1. Video of the time-lapse imaging described in Fig. 4a showing acidic streamers downstream of microcolonies in flow biofilms.

Supplementary Movie 2. Movie of the 3D rendering described in Fig. 5b showing acidic core of microcolonies in flow biofilms.
